# Oleyl group-functionalized insulating gate transistors for measuring extracellular pH of floating cells

**DOI:** 10.1080/14686996.2016.1198217

**Published:** 2016-07-26

**Authors:** Yuki Imaizumi, Tatsuro Goda, Yutaro Toya, Akira Matsumoto, Yuji Miyahara

**Affiliations:** ^a^ Institute of Biomaterials and Bioengineering, Tokyo Medical and Dental University (TMDU), Tokyo, Japan

**Keywords:** Ion-sensitive field-effect transistors, self-assembled monolayer, biocompatible anchor for cell membrane, T lymphocytes, hemolysis, plasma membrane injury, 30 Bio-inspired and biomedical materials, 101 Self-assembly/Self-organized materials, 201 Electronics/Semiconductor/TCOs, 208 Sensors and actuators, 212 Surface and interfaces, 502 Electron spectroscopy

## Abstract

The extracellular ionic microenvironment has a close relationship to biological activities such as by cellular respiration, cancer development, and immune response. A system composed of ion-sensitive field-effect transistors (ISFET), cells, and program-controlled fluidics has enabled the acquisition of real-time information about the integrity of the cell membrane via pH measurement. Here we aimed to extend this system toward floating cells such as T lymphocytes for investigating complement activation and pharmacokinetics through alternations in the plasma membrane integrity. We functionalized the surface of tantalum oxide gate insulator of ISFET with oleyl-tethered phosphonic acid for interacting with the plasma membranes of floating cells without affecting the cell signaling. The surface modification was characterized by X-ray photoelectron spectroscopy and water contact angle measurements. The Nernst response of −37.8 mV/pH was obtained for the surface-modified ISFET at 37 °C. The oleyl group-functionalized gate insulator successfully captured Jurkat T cells in a fluidic condition without acute cytotoxicity. The system was able to record the time course of pH changes at the cells/ISFET interface during the process of instant addition and withdrawal of ammonium chloride. Further, the plasma membrane injury of floating cells after exposure by detergent Triton™ X-100 was successfully determined using the modified ISFET with enhanced sensitivity as compared with conventional hemolysis assays.

## Graphical abstract

**Figure:**
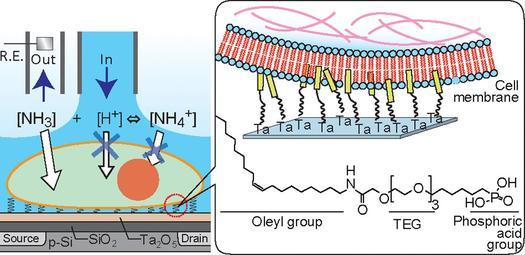

## 1. Introduction

Biological pH is heterogeneously distributed *in vivo*. For example, gastric mucosa generates a pH gradient for preventing gastric epithelial cell damage by gastric acid (pH 1.0–1.5).[1] Cell metabolism also alters extracellular pH. Cancer or tumor cells induce tissue acidification (pH 6.5–6.9), which is attributed to the secretion of lactic acid because of an enhanced glycolysis in anaerobic conditions.[2] Tissue acidification activates phagocytosis of leukocytes in the innate immune system.[3] The complement system is enhanced in acidic microenvironments.[4] Extracellular pH is closely related to intracellular activities such as by enzymes, ion channels, and organelles.[5] Fluorescence imaging by molecular probes or quantum dots is the standard method for pH sensing *in vitro* and *in vivo.*[6,7] Optical methods can be applied to individual cells due to a high spatiotemporal resolution. However, fluorescent probes suffer from photobleaching for long-term measurements. Occasionally, quantum dots are cytotoxic and are difficult to deliver to sites of interest. Potentiometric pH-sensing using a microelectrode is widely used.[8] These are label-free, but have limitation in positioning and miniaturization.

Previously, pH-responsive ion-sensitive field-effect transistors (ISFETs) have been used for non-invasive and label-free evaluation of cell metabolism, or the activity of ion channels.[9–14] The measurement of cell metabolism using ISFET is not suitable for acquiring pH at high temporal resolution because the system requires the medium to be flushed every few minutes to determine the acidification rate. Recently, we have developed an ISFET system for measuring the activity of membrane transporters expressed on a Xenopus oocyte.[12,13] We have further attempted to detect the ion barrier property of plasma membranes using ISFET by exposing cells to NH_4_Cl.[15] Forming a dense monolayer of adhesive cells enabled the pH measurement confined in the cells/ISFET interspace (60–80 nm).[14] Integrin proteins on plasma membranes are responsible for cell adhesion by interacting with an extracellular matrix coated on a gate insulator.[16] The cells/ISFET assay using NH_4_Cl showed enhanced sensitivity to the membrane disorder compared to the conventional hemolysis assay. However, our previous system is unable to apply to floating cells because floating cells lack some types of integrin that are responsible for interactions with the extracellular matrix on the plasma membrane.

There are several methods for capturing floating cells at desired positions on a material surface. First, microfluidic technologies are used for physically capturing individual cells under continuous flow.[17] Air pressurization can settle giant floating cells on a sensor surface. Optical tweezers manipulate each cell with an accurate force resolution without contact force.[18] These physical methods require no anchoring ligand or receptor. On the other hand, a rigorous arrangement of the distance between the sensor surface and the cell membranes is not possible. Physical stress might also change the cell activity.[19] The second strategy is to capture floating cells through anchor molecules such as cholesterol and glycosylphosphatidylinositol expressed on plasma membranes of floating cells.[20] However, the capturing efficiency depends on the composition of the plasma membrane of each cell type and the specific recognition may change the cell metabolism. In this study, we used a lipid anchoring molecule for capturing floating cells onto the gate insulator of ISFET. A lipid-mimetic oleyl chain is easily inserted into the hydrophobic core of plasma membranes without cell signaling or acute cytotoxicity.[21] We aimed to capture model T lymphocytes, Jurkat T cells, on an oleyl group-modified ISFET. Then, we attempted to measure pH at the floating cells/ISFET interface, as we have succeeded in the system using adhesive cells. Furthermore, we focused on the measurement of ion barrier breakdown of plasma membranes on exposure to detergent.

## 2. Material and methods

### 2.1. Materials

Jurkat T cell line was bought from Summit Pharmaceuticals International (Tokyo, Japan). Defibrinated fresh sheep blood was purchased from Nippon Bio-test Laboratories (Tokyo, Japan). Ta_2_O_5_ deposited SiO_2_ substrates (10 × 10 mm^2^, thickness: 300 Å) were purchased from Nanotechnology Innovation Station at NIMS (Tsukuba, Japan). N-channel ISFET with a 40 nm-thick Ta_2_O_5_ gate insulator was obtained from ISFETCOM (Saitama, Japan). Milli-Q water (EMD Millipore Co. Billerica, MA, USA) was used throughout the study. All the other reagents were from commercial sources and were used as received unless otherwise stated.

### 2.2. Self-assembly protocols

A glass tube (5 mm in inner diameter) was fixed on the top of the ISFET chip with thermosetting epoxy resin (120 °C, 2 h) to make a small chamber for cell culture. A rectangular Ta_2_O_5_ gate insulator (10 μm × 340 μm) was cleaned in a detergent solution with ultrasonication for 5 min, followed by a wash with water and 2-propanol for 15 min, three times. Ta_2_O_5_ substrates for X-ray photoelectron spectroscopy (XPS) were cleaned in 2-propanol with ultrasonication for 15 min, followed by plasma treatment at 300 W in O_2_/Ar for 1 min.

A self-assembled monolayer (SAM) was formed on the surface of Ta_2_O_5_ by dipping in 500 μmol l^−1^ carboxyl triethylenglycol hexylphosphonic acid (CEP) in 100:1 *n*-hexane/2-propanol (v/v) for 48 h at room temperature. Thereafter, the CEP-coated substrate was incubated in air at 120 °C overnight and was rinsed with 2-propanol. Next, the CEP SAM on Ta_2_O_5_ was reacted in a 1.3 μmol l^−1^ oleylamine (OA) in methanol with 0.17 mg ml^−1^ 4-(4,6-dimethoxy-1,3,5-triazin-2-yl)-4-methylmorpholinium chloride *n*-hydrate (DMT-MM) at 50 °C for 4 h to form oleylacetamide triethylenglycol hexylphosphonic acid (OEP) SAM, followed by a rinse in methanol.

### 2.3. X-ray photoelectron spectroscopy

The surface elemental composition was determined by XPS (AXIS-HSi165; Shimadzu-Kratos, Kyoto, Japan) equipped with a 15.0 kV Mg Kα radiation source at the anode. The take-off angle of the photoelectrons was set at 90.0°. The curve fitting of the high-resolution spectra was performed by using Gaussian functions. The mean and standard deviation were obtained from the measurements at three positions.

### 2.4. Water contact angle measurements

Surface wettability was measured by the sessile drop method using a contact angle measuring system (DM–501, Kyowa Interface Science, Saitama, Japan). A water droplet of 1.0 μl was placed on a planar surface. The static water contact angle at the liquid–vapor interface was determined by the image.

### 2.5. Cell culture

Jurkat T cells were cultured in a 25-cm^2^ non-treated flask in Roswell Park Memorial Institute (RPMI) medium supplemented with 10% fetal bovine serum (FBS), 1% GlutaMAX™-1, penicillin/streptomycin (100 μg ml^−1^) at 37 °C in 5% CO_2_ atmosphere.

### 2.6. Cell staining

The nuclei of Jurkat T cells (5 × 10^6^ cells ml^−1^) were stained by 10 μg ml^−1^ Hoechst 33342 in RPMI medium for 30 min at 37 °C in 5% CO_2_. The supernatant was removed by centrifugation at 1500 rpm for 10 min at 4 °C, and the isolated cells were washed with Dulbecco’s phosphate-buffered saline (DPBS). After removing DPBS by centrifuge, cytoplasm was stained in 1.8 μg ml^−1^ calcein-acetoxymethyl (calcein-AM) in DPBS for 30 min at 37 °C in 5% CO_2_. After removing the calcein-AM solution by centrifuge, the stained cells (1 × 10^6^ cells ml^−1^) in DPBS were seeded onto the OEP SAM at 37 °C in 5% CO_2_. The captured cells were observed by fluorescence microscope (Nikon ECLIPSE FN1, Tokyo, Japan). Bright field images of the cells on ISFET were taken by upright microscope (Olympus SZX12, Tokyo, Japan).

### 2.7. ISFET system for measuring pH at cells/gate insulator interspace

The ISFET was connected to a home-built analyzer/data logger. The ISFET was operated at the drain-source current of 0.5 mA at the drain-source voltage of 0.5–1.0 V at no DC bias for the Ag/AgCl pellet reference electrode in the buffer solution. Then, Jurkat T cells (1 × 10^6^ cells) in RPMI medium were seeded on the gate insulator and were incubated for 30 min prior to the measurements.

A bis-tris-propane (BTP) buffer (1 mmol l^−1^ BTP, 140 mmol l^−1^ NaCl, 4 mmol l^−1^ KCl, 1 mmol l^−1^ MgCl_2_ and 20 mmol l^−1^ sucrose, pH 7.4) was used for conditioning and rinsing during the measurements. The BTP buffer containing 20 mmol l^−1^ NH_4_Cl instead of 20 mmol l^−1^ sucrose (pH 7.2) was used for quickly modulating intra/extra-cellular pH. The NH_4_Cl-loaded/unloaded buffers were alternately subjected to the cells on the OEP SAM-modified gate insulator in a stepped manner using a program-controlled perfusion system at 40–60 μl min^-1^ at 37 °C. The vertical distance between the gate insulator and the outlet of infusing tube was about 80 μm for instant exchanges of the solutions surrounding cells. For evaluating plasma membrane damage, a detergent Triton^™^ X-100 (TX-100) was exposed to the cells on ISFET using the perfusing system during the intervals of NH_4_Cl unloading for 1 min.

Time-course of pH changes (Δ*pH*) was measured from changes in the gate voltage (Δ*V*_*G*_) by the Nernst equation:

(1) $$\Delta V_{G}=2.303\left(RT/nF\right)\Delta \log\left[H^{+}\right]=-2.303\left(RT/nF\right)\Delta pH$$

where the constant −*2.303 (RT/nF)* corresponds to −59.2 mV at 25 °C.[22]

### 2.8. Hemolysis assay

Plasma components and buffy coat in defibrinated fresh sheep blood were removed by centrifugation at 1500 × *g* for 5 min. The isolated erythrocytes were washed three times with isotonic tris-buffered saline (1 × TBS, pH7.4). Four volumes of washed and packed erythrocytes were diluted with three volumes of TBS to produce a 57% hematocrit solution. Then, 75 μl of TBS with the desired concentration of TX-100 was added to 175 μl of the erythrocyte solution (final hematocrit: 40%), followed by incubation on ice for 20 min. Thereafter, the supernatant was isolated by centrifugation at 1500 × *g* for 5 min at 4 °C. The degree of hemolysis was determined from the absorbance at 543 nm using a microplate reader (infiniteM200, TECAN, Manndorf, Switzerland). Controls were prepared by treating the supernatant from the erythrocytes with water (i.e. 100% hemolysis) and TBS (i.e. 0% hemolysis).

### 2.9. Quantification of solubilized phospholipids

Erythrocytes incubated on ice were separated and washed in 1 ml TBS. After removing TBS by centrifuge, 200 μl TBS was added and the erythrocyte was destroyed by centrifuge at 16,500 g at 4 °C for 30 min to obtain an unsolubilized pellet. An amount of 770 μl of 2-propanol was added to vortex for 1 h at room temperature, followed by adding 490 μl of chloroform to vortex for 1 h. The suspension was centrifuged at 2000 g for 10 min and the supernatant as a water phase was replaced. The clear organic layer was collected, followed by adding 500 μl of TBS. A 500 μl aliquot of 2:1 methanol-chloroform (v/v) was added for mixing. After centrifuging at 15,000 × *g* at 4 °C for 5 min, the supernatant was replaced and the clear organic layer was separated and evaporated to yield a dry lipid extract. Phospholipids (PLs) in the dry extract were quantified by inorganic phosphorus assay. The extract was treated by 500 μl of 0.1 mol l^−1^ sulfuric acid and 25 mg of sodium peroxodisulfate, followed by autoclaving at 123 °C for 30 min. After cooling, the solution was neutralized by adding 500 μl of 0.2 mol l^−1^ NaOH. Then, to the sample was added 800 μl of water, 100 μl of 1.25% ammonium molybdate in water, and 100 μl of 5% ascorbic acid in water for incubation at room temperature for 30 min. The amount of total phosphorus from unsolubilized PLs was determined by the absorbance at 880 nm using a microplate reader.

## 3. Results and discussion

### 3.1. Capturing floating cells on OEP SAM-modified surfaces

**Figure 1.**
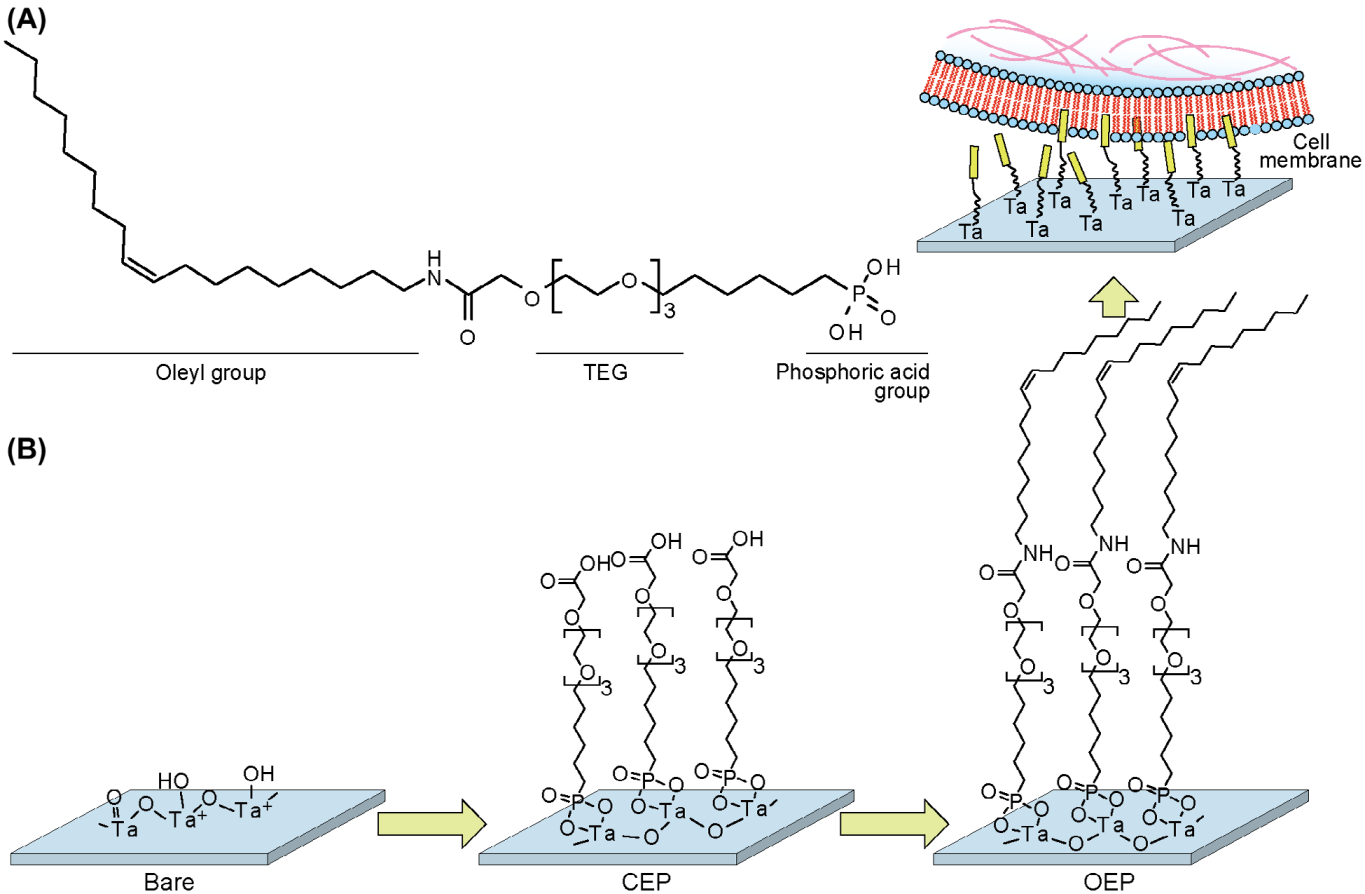
Capturing floating cells on the oleyl-modified ISFET. (A) Chemical structure of oleylacetamide triethylenglycol hexylphosphonic acid (OEP). (B) Capturing of Jurkat T cells on OEP SAM-modified ISFET. OEP SAM was constructed on the gate insulator by two-step reaction. The CEP SAM was formed by phosphonic acid ester bond, followed by amide condensation between CEP and OA. The oleyl group was anchored onto the hydrophobic core of plasma membranes.

OEP is consisted of hydrophobic oleyl group, hydrophilic triethylene glycol (TEG) linker, and metal oxide-reactive phosphonic acid (Figure 1(A)). The oleyl group can be inserted into the lipid cores of plasma membranes.[20] This strategy is effective for capturing floating cells without preferential interaction between the cells and material surfaces via proteins or sugar chains. The OEP SAM was constructed *in situ* on the Ta_2_O_5_ gate insulator via two-step reaction (Figure 1(B)). First, the phosphonic acid group was covalently attached onto the hydroxide groups on the surface of Ta_2_O_5_ for making CEP SAM.[23] Second, the amino group of OA was reacted with the carboxyl group of CEP using a catalyst DMT-MM.[24] Floating T lymphocytes, Jurkat T cells, were simply seeded onto the OEP SAM-modified surface.

### 3.2. Surface characterization

**Figure 2.**
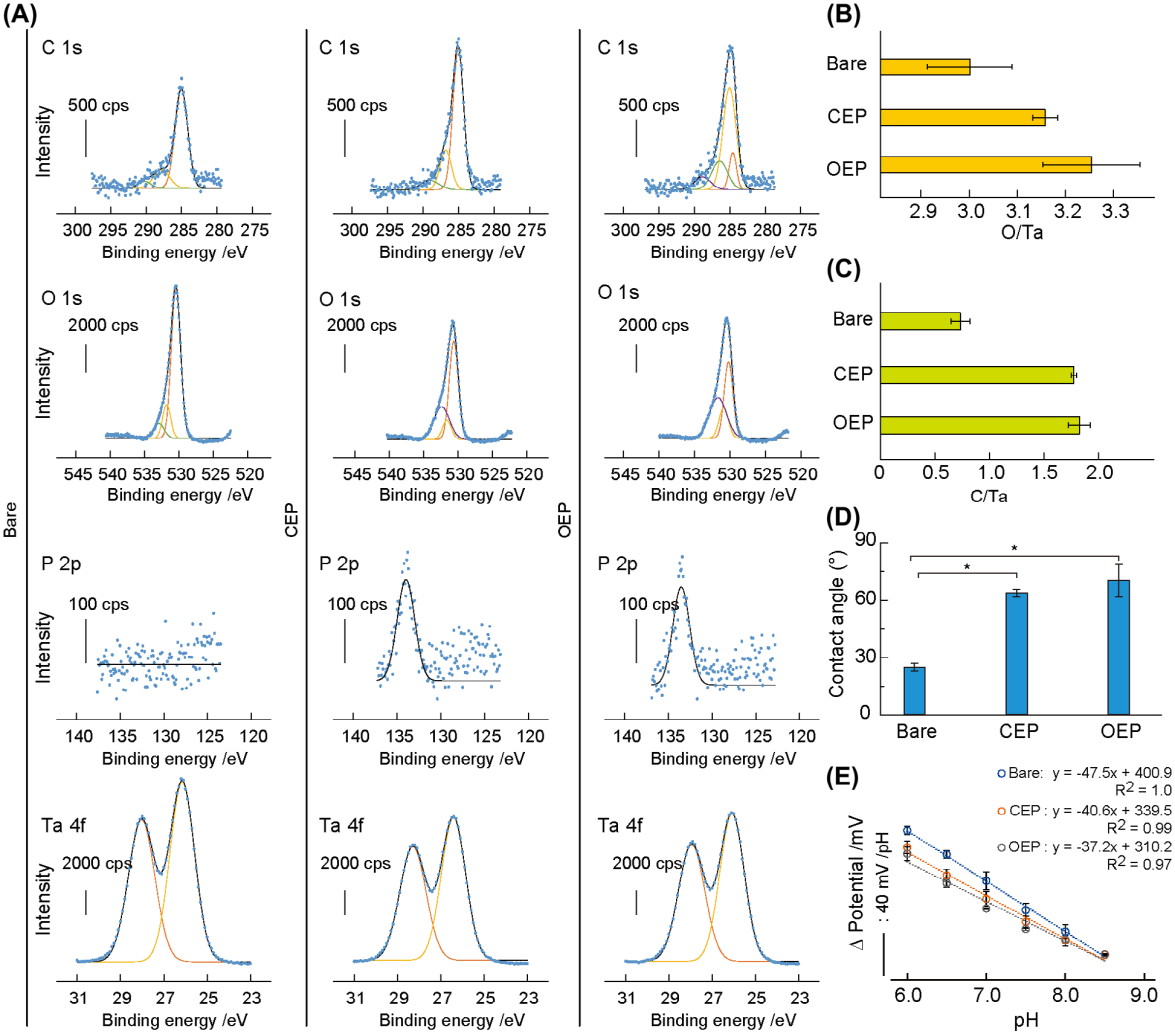
Surface characterization. (A) High-resolution C 1s, O 1s, P 2p, and Ta 4f XPS spectra on the surface of bare Ta_2_O_5_, CEP SAM, and OEP SAM. Solid lines indicate curve fitting results. (B, C) The O/Ta (B) and C/Ta (C) atomic ratios on the bare Ta_2_O_5_, CEP SAM, and OEP SAM surfaces (*n* = 3). (D) Static water contact angles on the bare Ta_2_O_5_, CEP SAM, and OEP SAM surfaces. Data are represented as mean ± standard deviation (*n* = 3).*p < 0.05. (E) The Nernst responses on the bare Ta_2_O_5_, CEP SAM, and OEP SAM surfaces. Data are represented as mean ± standard deviation (*n* = 3).

The elemental composition of each surface was determined by XPS (Figure 2(A)). High-resolution C 1s spectra identified the C–C bond at 285.0 eV and the C–O bond at 286.4–287.8 eV on the bare Ta_2_O_5_, CEP SAM, and OEP SAM surfaces. The O=C–O bond was observed at 290.2 eV on the bare surface and at 289.0 eV on CEP SAM. The C=C bond appeared at 284.6 eV on OEP SAM. The O–C–NH bond was measured at 288.8 eV on OEP SAM. High-resolution O 1s spectra identified the peak corresponding to Ta_2_O_5_ at 530.4 eV on the bare surface, CEP SAM, and OEP SAM. The C–O–C bond was detected at 532.4 eV on CEP SAM and OEP SAM. The –OH peak at 531.8 eV and the H_2_O peak at 532.9 eV were observed on the bare surface. There was the P=O bond at 531.6 eV on CEP SAM and the C=O bond at 530.9 eV on OEP SAM. High-resolution P 2p spectra identified the peak of phosphonic acid ester bond at 134.0 eV on CEP and OEP SAMs. High-resolution Ta 4f spectra identified Ta_2_O_5_ at 26.2 eV on all surfaces.

The atomic ratio of O/Ta and C/Ta were calculated from the peak areas of each element (Figure 2(B)). The O/Ta ratio increased by the SAM formation on the Ta_2_O_5_ surface by each step because of the presence of O atoms in CEP and OEP molecules. The C/Ta ratio increased by 2.4-fold and 2.5-fold following the formation of CEP and OEP SAMs, respectively.

Surface wettability of bare Ta_2_O_5_, CEP SAM, and OEP SAM was determined by water contact angle measurements based on the Young equation.[25] The contact angle increased from 24.8° to 63.9° and 70.5° with significance (*n* = 3, *p* < 0.05) by forming the CEP and OEP SAMs. The increased hydrophobicity was due to the presence of alkyl chains on these SAMs.

Because the hydroxide group on the pristine Ta_2_O_5_ gate insulator is responsible for the pH response,[22] the sensitivity of the ISFET was checked after the modification of the gate insulator with CEP and OEP SAMs. The Nernst response was −47.5 ± 1.4 mV/pH for the original ISFET, −40.6 ± 2.1 mV/pH for the CEP SAM-formed ISFET, and −37.2 ± 2.7 mV/pH for the OEP SAM-formed ISFET, respectively (*n* = 3), at the pH ranges of 6.0–8.5 at 37 °C. The decreased sensitivity is attributed to the consumption of the –OH groups by phosphonic acid ester linkage for making SAM. Nevertheless, the OEP SAM-modified ISFET can be used for measuring pH at the cells/ISFET interspace.

### 3.3. Cell capturing

**Figure 3.**
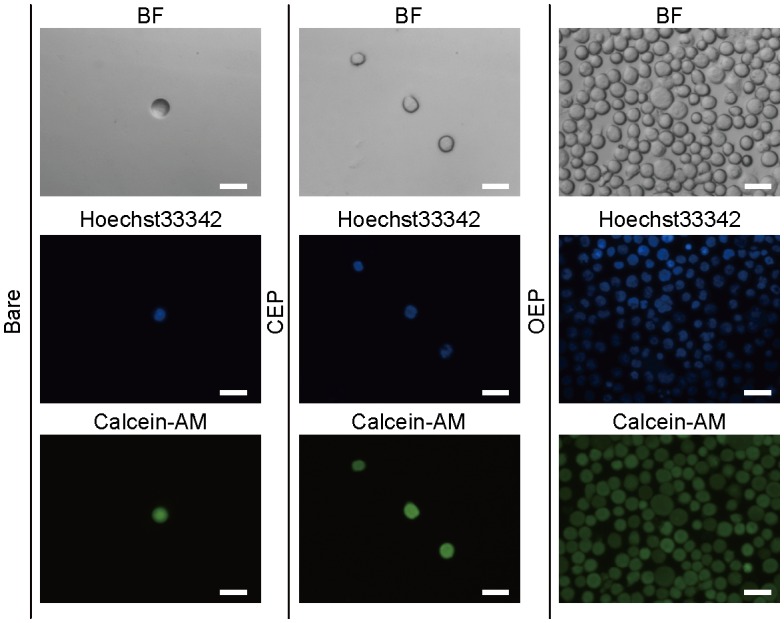
Images of Jurkat T cells on the bare Ta_2_O_5_, CEP SAM, and OEP SAM surfaces. BF: bright field. Cell nucleus and cytoplasm were stained by Hoechst 33342 and calcein, respectively. Scale bars: 20 μm.

The insertion of oleyl group in the hydrophobic core of plasma membranes for immobilization of Jurkat T cells might cause membrane injury or acute cytotoxicity. To check the cell viability, nucleus and cytosol were stained with Hoechst 33342 and calcein, respectively. The cells seeded on the bare Ta_2_O_5_, CEP SAM, and OEP SAM surfaces were observed with a fluorescence microscope (Figure 3). The cells were securely adhered on the OEP SAM surface, but not on the surfaces of bare Ta_2_O_5_ or CEP SAM. Hoechst 33342 staining showed no fragmentation of the nucleus, indicating no sign of apoptosis induced by genotoxic activity.[26] The fluorescence by calcein, which is impermeable to healthy plasma membranes, in the cytoplasm indicates that the insertion of oleyl group cause no leakage of the indicator across the OEP-contained plasma membrane.[27]

### 3.4. Ammonium chloride-induced pH oscillations

**Figure 4.**
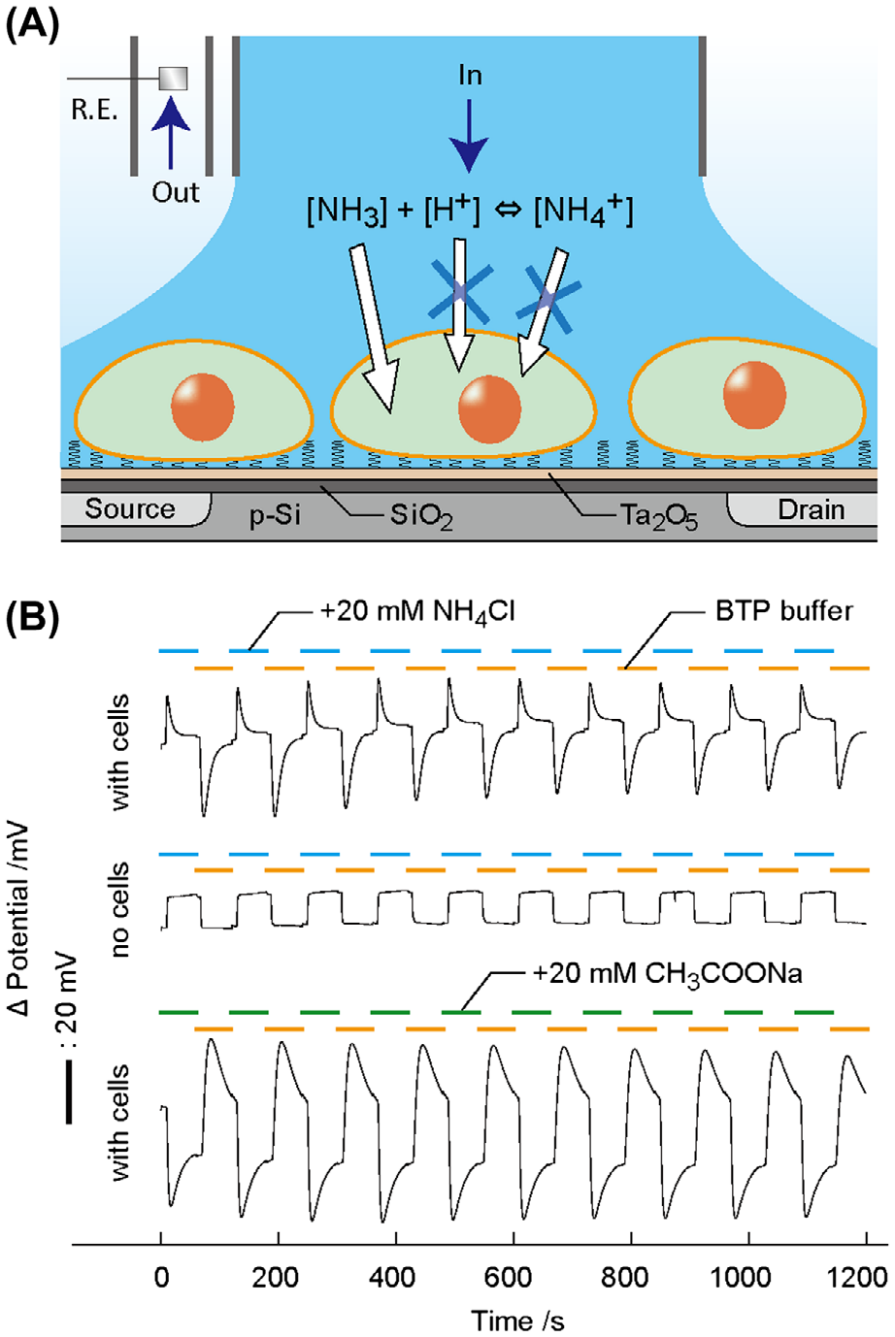
Stepped input of weak acid or base generated pH transients at the cells/ISFET interspace. (A) Jurkat T cells were cultured on the OEP SAM-modified ISFET. Continuous perfusion system allowed instant exchanges of isotonic buffers with or without NH_4_Cl surrounding the cells. The intervals of NH_4_Cl loading/unloading induced pH transient at the cells/ISFET interface. The Ag/AgCl pellet used as a reference electrode (R.E.). (B) Time course of the ISFET potential during alternate injections of isotonic buffers with or without 20 mmol l^−1^ NH_4_Cl for 60 s each.

The Jurkat T cells-immobilized ISFET sensor in conjunction with a perfusion system was used for measuring pH in potentiometry (Figure 4(A)). To manipulate pH in the cell microenvironment instantly, we introduced a weak acid or base. NH_4_Cl is used in physiology to alter pH in cytosol via the proton sponge effect without affecting cell metabolism.[28] The pH changed transiently by 0.8 units at the point of NH_4_Cl loading and unloading in a stepped manner (Figure 4(B)). The pH change is reproducible during the intervals of NH_4_Cl addition/withdrawal for 10 times with the relative standard deviation (RSD) of 6.9%. This indicates that the NH_4_Cl treatment does not affect cell viability. The pH response by the solution exchange is attributed to the ion-barrier property of healthy plasma membranes. At the point of NH_4_Cl addition, neutral NH_3_ passively diffuses into the cytoplasm across the plasma membrane, resulting in the generation of a temporary excess of H^+^ in the close proximity of outer cell membranes by balancing the ammonia equilibrium. After the completion of NH_3_ loading into the cytoplasm, the time course of the signal returned to a stable pH from the buffer solution. When NH_4_Cl was withdrawn from the bulk solution, NH_3_ in the cytoplasm passively diffused out across the plasma membranes, leading to the consumption of H^+^ in the close proximity of the outer cell membrane for rebalancing the NH_4_^+^/NH_3_ equilibrium. After the NH_3_ release was complete, the signal returned to the original pH. The characteristic pH changes only occurred in the presence of cells on ISFET. Without cells, the potential exhibited the pH values from the two infused buffers during the intervals. The proposed mechanism was supported by the treatments of sodium acetate (CH_3_COONa) instead of NH_4_Cl, which showed the opposite trend in pH during the stepped addition/withdrawal of CH_3_COONa. The opposing directions are explained by the absorption of H^+^ for generating membrane-permeable CH_3_COOH and the production of H^+^ for generating membrane-permeable NH_3_. The magnitude of pH changes was different between CH_3_COONa and NH_4_Cl, which is derived from a lower pKa of 4.76 for acetate compared with ammonium (pKa = 9.25) at 37 °C.[29,30] Namely, the ability of proton generation is higher in CH_3_COOH than NH_4_^+^.

### 3.5. Investigating the ion-barrier function of plasma membrane

**Figure 5.**
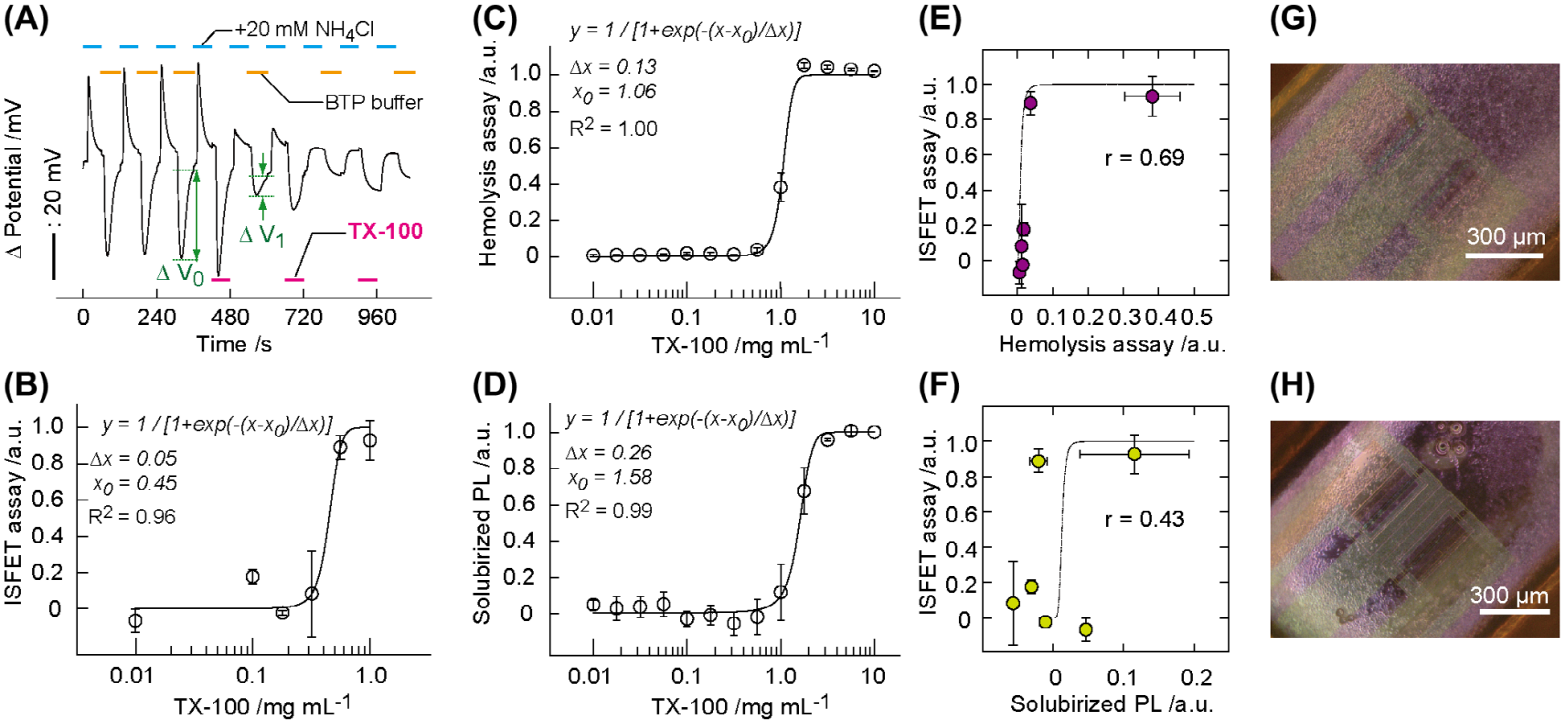
A decrease in pH is correlated to plasma membrane damage. (A) Changes in the ISFET signal (1 − Δ*V*/Δ*V*_0_) of the ISFET assay was used as a parameter for membrane injury. Δ*V* was obtained after exposing the cells to TX-100 at desired concentrations for 60 s. (B) The ISFET signal after exposing the cells to TX-100 at desired concentration for 60 s. The solid line and equation represent the curve fitting result using the logistic function. Data are presented as mean ± standard deviation (*n* = 3). (C, D) The degree of hemolysis (C) and solubilized phospholipid (PL) (D) of sheep erythrocytes after incubating in TX-100 for 20 min (*n* = 3). (E, F) The scattered plot showing the correlation between the ISFET signal and the degree of hemolysis (E) and between the ISFET signal and the degree of solubilized PL (F) for TX-100 exposure to the cells. The solid line shows the standard curve obtained by the logistic function for each assay. Correlation coefficients: r = 0.69 (E) and 0.43 (F). Data are represented as mean ± standard deviation (*n* = 3). (G, H) BF micrographs of the cells on ISFET after treated with 0.0 mg ml^−1^ (G) or 1.0 mg ml^−1^ (H) TX-100.

Because the pH changes were derived from the ion barrier properties of healthy plasma membranes, we intended to evaluate the membrane injury of Jurkat T cells on challenge by a membrane-toxic detergent. An irreversible decrease in the pH transient was observed with repeated exposure of the cells to 1 mg ml^−1^ detergent TX-100 during the NH_4_Cl intervals (Figure 5(A)). The decrease in the pH transients indicates the cancellation of temporary imbalanced equilibrium of NH_4_^+^/NH_3_ because of free permeation of NH_4_^+^ and H^+^ as well as NH_3_ across the damaged plasma membranes. The reduction ratio after the first exposure for 1 min (1−Δ*V*_1_ */*Δ*V*_0_) was used as a parameter for the membrane injury. The ISFET signal was concentration-dependent on TX-100 (Figure 5(B)). The damage of plasma membrane following a TX-100 exposure was further evaluated using the hemolysis and solubilized phospholipids (PLs) assay as standard techniques.[31] Trends were similar between the hemolytic activity and the amount of solubilized PLs on challenge by TX-100 (Figure 5(C) and 5(D)). The concentration dependence of these values was fitted by the logistic function.[31]

(2) $$y=1/[1+\exp\left(-\left(x-x_{0}\right)/\Delta x\right)]$$

where *x*_0_ is the detergent concentration at 50% value for each assay and was 0.45, 1.06, and 1.58 mg ml^−1^ for the ISFET, hemolysis, and solubilized PL assays, respectively. Δ*x* is the scale parameter of the sigmoid transition and was 0.05, 0.13, and 0.26 mg ml^−1^ for the ISFET, hemolysis, and solubilized PL assays, respectively. By extrapolating the straight portion of the curve to the lower concentrations, we evaluated the characteristic concentration (*x* ^*on*^ = *x*_0_−2Δ*x*) corresponding to the onset of plasma membrane injury. The *x* ^*on*^ value was 0.35, 0.80, and 1.06 mg ml^−1^ for the ISFET, hemolysis, and solubilized PL assays, respectively. The comparison of *x*_0_ or *x* ^*on*^ values indicates that the ISFET assay is sensitive by several-fold compared with the hemolysis and PL assays in the detection of plasma membrane disordering. The high sensitivity is attributed to the small sizes of H^+^ and NH_4_^+^ (hydrodynamic radii ≤ 0.33 nm) [32] as compared with hemoglobin (hydrodynamic radii > 3.1 nm) [33] in the hemolysis assays. By considering the critical micelle concentration (CMC) of 0.16 mg ml^−1^ for TX-100,[34] TX-100 as a micelle is the cause of pore-forming activity onto plasma membranes in a concentration-dependent manner. At low concentrations, TX-100 micelles disturb plasma membranes by creating pores that are accessible for low-molecular-weight electrolytes. At intermediate concentrations, relatively large pores are formed on the plasma membrane and hemoglobin proteins are leaked through the large pores from the cytosol. At high concentrations, PLs as constituent molecules of plasma membranes are dissolved into the solution phase by forming a complex with TX-100 micelles. The comparison of the ISFET signal with these results in the scattered plots indicates the correlation between the ISFET assay with the erythrocyte-based assays with the factor of 0.43–0.69 (Figure 5(E) and 5(F)). The high ISFET signals at the nearly zero values from the hemolysis and solubilized PL assays at 0.1–1 mg ml^−1^ TX-100 indicate the enhanced sensitivity of the ISFET assay for measuring the injury of plasma membranes. Bright field micrographs showed lysis of cells on ISFET after in contact with 1.0 mg ml^−1^ TX-100 (Figure 5(G) and 5(H)).

## 4. Conclusions

We developed OEP SAM for capturing floating T lymphocyte cells on the Ta_2_O_5_ gate insulator. The OEP SAM-modified ISFET maintained the pH sensitivity and captured floating cells under the shear stress of the continuous flow without acute cytotoxicity. The floating cells/ISFET system achieved real-time pH monitoring during the intervals of NH_4_Cl addition/withdrawal. The instant exchange of NH_4_Cl induced the reproducible pH transients because of the ion barrier property of the intact plasma membrane. The cell/ISFET system was applied for detecting ion-accessible pores on the plasma membranes of Jurkat T cells on challenge by detergent TX-100. The enhanced sensitivity is responsible for the small sizes of NH_4_^+^ and H^+^ indicators for measuring the membrane toxicity of exogenous compound. The OEP SAM-modified ISFET system would extend the availability of the pH-based assay toward the measurements of bioactivities using floating cells.

## Disclosure statement

No potential conflict of interest was reported by the authors.

## Funding

T.G. is grateful for financial support from the Grant-in-Aid for Scientific Research on Innovative Areas ‘Nanomedicine Molecular Science’ [#26107705] from MEXT of Japan and the Nakatani Foundation of Electronic Measuring Technology Advancement and Ministry of Education, Culture, Sports, Science, and Technology [26107705].
